# Sol-Gel Synthesis of the Double Perovskite Sr_2_FeMoO_6_ by Microwave Technique

**DOI:** 10.3390/ma14143876

**Published:** 2021-07-12

**Authors:** Jesús Valdés, Daniel Reséndiz, Ángeles Cuán, Rufino Nava, Bertha Aguilar, Carlos M. Cortés-Romero, Oracio Navarro

**Affiliations:** 1 División de Estudios de Posgrado, Facultad de Ingeniería, Centro Universitario, Universidad Autónoma de Querétaro, Querétaro 76010, Mexico; jesus.valdes@uaq.mx (J.V.); daniel.m.resendiz@gmail.com (D.R.); rufino@uaq.mx (R.N.); 2 Unidad Morelia, Instituto de Investigaciones en Materiales, Universidad Nacional Autónoma de México, Antigua carretera a Pátzcuaro No. 8701, Col. Ex Hacienda de San José de la Huerta, Morelia 58190, Mexico; baguilar@iim.unam.mx (B.A.); navarro@unam.mx (O.N.); 3 Facultad de Química, Centro Universitario, Universidad Autónoma de Querétaro, Querétaro 76010, Mexico; carlos.cortes@uaq.mx

**Keywords:** structural ordering, microwave heating, curie temperature, hydrothermal, crystal size

## Abstract

The effect of microwave radiation on the hydrothermal synthesis of the double perovskite Sr_2_FeMoO_6_ has been studied based on a comparison of the particle size and structural characteristics of products from both methods. A temperature, pressure, and pH condition screening was performed, and the most representative results of these are herein presented and discussed. Radiation of microwaves in the hydrothermal synthesis method led to a decrease in crystallite size, which is an effect from the reaction temperature. The particle size ranged from 378 to 318 nm when pH was 4.5 and pressure was kept under 40 bars. According to X-ray diffraction (XRD) results coupled with the size-strain plot method, the product obtained by both synthesis methods (with and without microwave radiation) have similar crystal purity. The Scanning Electron Microscopy (SEM) and Energy Dispersive X-ray Spectroscopy (EDS) techniques showed that the morphology and the distribution of metal ions are uniform. The Curie temperature obtained by thermogravimetric analysis indicates that, in the presence of microwaves, the value was higher with respect to traditional synthesis from 335 K to 342.5 K. Consequently, microwave radiation enhances the diffusion and nucleation process of ionic precursors during the synthesis, which promotes a uniform heating in the reaction mixture leading to a reduction in the particle size, but keeping good crystallinity of the double perovskite. Precursor phases and the final purity of the Sr_2_FeMoO_6_ powder can be controlled via hydrothermal microwave heating on the first stages of the Sol-Gel method.

## 1. Introduction

The general formula of an ideal double perovskite is A_2_B’B’’O_6_, where A denotes an alkaline-earth or rare-earth ion, B’ and B’’ are transition-metal sites, and there are oxygen bridges every B’ and B’’, which gives an alternating B’O_6_ and B’’O_6_ octahedral form. The double perovskite Sr_2_FeMoO_6_, known as SFMO, consist of a body centered cubic lattice with alternating FeO_6_ and MoO_6_ octahedral at the corners and the strontium atom in its center [1]. SFMO compound is a half-metallic ferromagnetic oxide with colossal magnetoresistance and a Curie temperature of 400 K [2]. This transition metal oxide has been widely investigated in view of their attractive applications for spintronics and digital storage [3]. Recently [4], the development of double perovskite as a catalyst has been explored in various applications including general catalysis, electrocatalysis, and photoelectrocatalysis. Particularly, one of the best opportunities in energy storage and eco-friendly applications is related to the double perovskite as photocatalyst. As the most plentiful renewable energy source is sunlight, it can be converted into chemical energy stored in fuels or chemicals, either through a direct approach (via photoelectrochemical processes) or indirect one (via solar-assisted electrochemical processes). Such fuels or chemicals, when fed into electrochemical devices (fuel cell or batteries), can be used to produce electrical energy, powering our planet with a reduced carbon footprint compared to traditional fossil fuels.

Traditionally, SFMO synthesis is performed by the solid-state reaction, that is, calcination and reduction in initial reactants, usually SrMoO_4_ and SrFeO_3−x_. This method requires a high-energy reactor working at 1700 rpm for about 5 h; final morphology and crystallite size control are difficult to achieve [5]. The latter two characteristics might affect the magnetoresistance and half-metallic behavior of the SFMO [6,7]. Morphology and crystallite size can be controlled by the Sol-Gel citrate technique; nucleation and mass transport require less energy than the Solid-State reaction, due to the homogeneity of the final gel [8]. The citric acid plays the role as a chelating agent, modifying the hydrolysis of the metal ions and forming a metal-citrate, the gelation process occurs with the inter-molecular bond of the metal-citrates, and can be tuned with pH and temperature. This leads to a problem; some cations could precipitate easily, leaving a narrow choice for pH control [9].

Recently, reports of SMFO demonstrated that the electronic properties of this material could be modulated depending on the synthesis method, pH, temperature, and pressure. For example, by generating a compound with high crystallinity that would have a potential application in the use of solar cells [10], or by varying the pH and using surfactant agents to generate a material with defects (oxygen vacancies) that can act as a good photocatalyst [11]. It should be mentioned that, in order to modulate structural and electronic properties, using characterization techniques such as XRD and derivative thermogravimetry analysis (DTG) is very important since this leads to a route towards achieving the product and determining the treatment conditions of the samples, such as calcination and reduction temperatures [12].

Depending on their chemical nature, some compounds in liquid or solid state have the characteristic of absorbing microwave radiation to raise their temperature [13]. This energy transfer is achieved from the dissipation of electromagnetic energy into heat, in contrast to conventional heating where heat transfer is slow and occurs from surface to bulk. Microwave-assisted synthesis allows selective absorption of electromagnetic radiation uniform heating, and even promotes reactions that usually do not occur under classical conditions [14,15,16]. In addition, heating a solution with microwaves in a closed container helps to develop a hydrothermal process; the high pressure and temperature in the container modifies the behavior of the chemical species in the solution and stimulate the generation of new phases [17,18,19].

The intergrain-magnetoresistance of polycrystalline SFMO depends on its particle size [20] and the amount of insulating nonmagnetic SrMoO_4_ impurity formed during preparation [21,22]. In addition, the cationic disorder breaks down the half-metallic ferromagnetic behavior [23,24]. Therefore, the synthesis method and conditions must be carefully selected in order to develop a better crystalline phase of SFMO, with a decrease in cationic disorder. Based on this fact, in the present work, a new experimental synthesis technique, namely the Sol-Gel microwave assisted synthesis method, was performed in order to obtain a high purity crystalline phase of SFMO. This material crystal structure, crystallite size and Curie temperature are compared to those of the traditional Sol-Gel and Solid-State methods. Special emphasis is placed on XRD and SEM analyses results that were correlated to the experimental procedures, either to the SFMO citrate Sol-Gel synthesis method, or that to citrate Sol-Gel method assisted by microwave. The apparent control on the crystallite size for the citrate Sol-Gel assisted by microwave method is inferred from size-strain plots.

## 2. Materials and Methods

### 2.1. Reagents

Strontium nitrate Sr(NO_3_)_2_ (99.995%, Aldrich, St. Louis, MO, USA), iron (III) nitrate nonahydrate Fe(NO_3_)_3_ 9H_2_O (99.95%, Aldrich, St. Louis, MO, USA), molybdenum (VI) oxide MoO_3_ (99.99%, Aldrich, St. Louis, MO, USA), citric acid monohydrate (99%, Aldrich, St. Louis, MO, USA) and ammonium hydroxide solution (28.0–30.0%, Aldrich, St. Louis, MO, USA) were used to form the SFMO precursor. The reagents were weighted according to their stoichiometric rate, molar ratio of 4.3:1 for citric acid and 2Sr + Fe + Mo salts were used in both methods, citrate Sol-Gel and the citrate Sol-Gel assisted by microwave.

### 2.2. Sol-Gel Method

Two experiments were performed by the normal sol-gel method using precursor metal citrates under optimal experimental conditions and were used as a reference, sol-gel experiment A (SGA) (pH = 2.5) and B (SGB) (pH = 4.5), for those in which microwave radiation was used. First, water was heated up to 80 °C under vigorous stirring with ammonium hydroxide solution to adjust the pH of the solution, and the corresponding mass of molybdenum (VI) oxide was added in order to obtain precursor substance ammonium molybdate. Subsequently, citric acid, strontium nitrate, and iron (III) nitrate nonahydrate were added according to their stoichiometric rate. The solution was kept with slow and laminar magnetic stirring at 80 °C, allowing water to be evaporated to half of the reaction volume solution. This mixture was dried for 24 h at 90 °C and, afterwards, the obtained gel was calcined for 3 h at 900 °C. Finally, the obtained powder was reduced at 1200 °C for 3 h in an Ar/5% H_2_ atmosphere.

### 2.3. Sol-Gel Method assisted by Microwave Radiation

The sol-gel afore-mentioned method using microwave radiation was performed for nine experiments. Similarly, the metallic precursor was obtained and, before the drying step, the reaction mixtures were placed in a microwave Synthos 3000 from AntonPaar, Graz, Austria, with a specific reaction time, temperature, and pH that are summarized in Table 1. Afterwards, in the current procedure, the resulting solutions were dried for 24 h at 90 °C and the obtained gel products were calcined for 3 h at 900 °C. Finally, the obtained powders were reduced at 1200 °C for 3 h in an Ar/5% H_2_ atmosphere.

### 2.4. X-ray Diffraction

The crystal structure and particle size of the precursor phases of SFMO powders and the SFMO final phase were investigated by X-ray diffraction. The crystallographic patterns of SFMO and its precursor phases were recorded with a D8 ADVANCE diffractometer (Blue Scientific, Cambridge, UK) from Bruker. For the powder diffraction, CuK_α1_ radiation with a wavelength of 1.5406 Å, a LiF crystal monochromator, and Bragg–Brentano diffraction geometry were used. The data were acquired at 25 °C, with a step-scan interval of 0.020° and step time of 5 s.

The two main properties extracted from the Bragg reflections-width are the crystallite size and the lattice strain. In cases where the diffracting domains are isotropic, and also a microstrain contribution exists, the size-strain plot (SSP) method is used, which has the advantage that less weight is given to reflections at high angles. This SSP method considers contributions to reflection broadening by considering anisotropy in network strain. This approach assumes that the crystallite size profile is described by a Lorentz function, while the tension in the lattice is described by a Gaussian function; the convolution of both functions describes the broadening of the reflections in a more precise way, as this method does not have a dependence on θ when calculating strain, compared to the Williamson–Hall method. The crystallite size is determined with the SSP method from the slope of the linearly fitted data and the root of the y-intercept gives the strain.

### 2.5. Scanning Electron Microscopy and Energy Dispersive X-ray Spectroscopy

The microstructure and topology of the SFMO powders were investigated by Scanning Electron Microscopy (JOEL, Tokyo, Japan). All the images were created using secondary electrons. The SEM analyses were performed using a JSM-IT300 from JEOL operated at 20.0 kV. The microscope has a double detector for the Energy Dispersive X-Ray Spectroscopy (EDS) (JOEL, Tokyo, Japan). This technique is used for chemical characterization of the samples, and for mapping the distribution of the elements.

### 2.6. Thermogravimetric Analysis and Differential Scanning Calorimetry

Curie temperature (Tc) was obtained by Thermogravimetric Analysis (TGA) and Differential Scanning Calorimetry (DSC). The DSC and TGA analyses were performed in a Q1000 and in a Q500, respectively, which are from TA Instruments (TA Instruments, New Castle, DE, USA).

To determine the Tc, the sample was placed in a TGA instrument, and brought up to 150 °C in a nitrogen atmosphere in order to dehydrate the sample, then cooled down to 25 °C and applied a magnetic field, using a magnet which was placed below the furnace and then heated to 300 °C to find the magnetic transition. Finally, this transition from ferromagnetic phase to paramagnetic phase determines the Curie temperature.

## 3. Results

### 3.1. X-ray Diffraction

**Figure 1:**
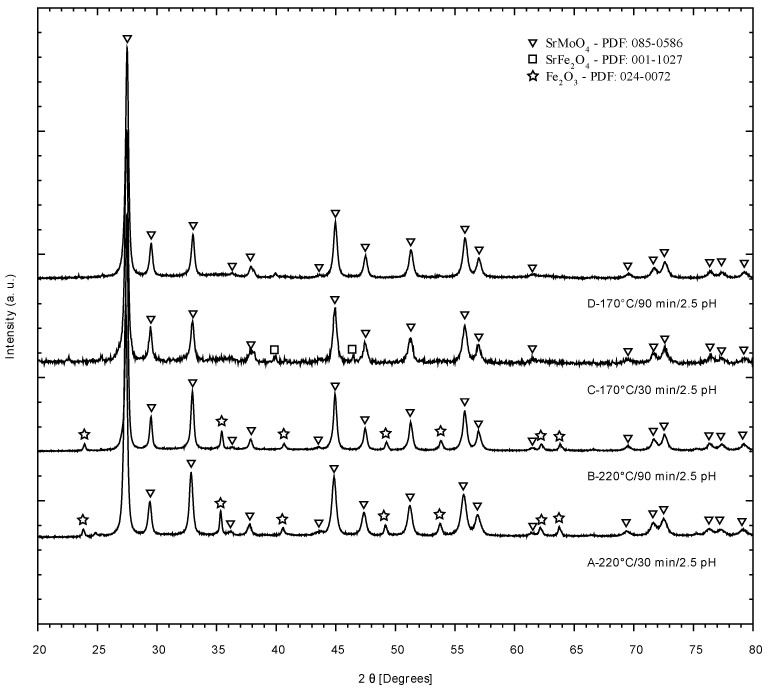
XRD patterns of the solid obtained in experiments A, B, C, and D in which microwave radiation was used. Bragg reflections attributed to main components (SrMoO_4_ (PDF: 085-0586) and Hematite, Fe_2_O_3_ (PDF: 024-0072)) are highlighted.

After radiation in experiments A, B, C, and D, a fine powder precipitate was obtained, dried, and analyzed by XRD. Figure 1 shows the XRD pattern for these four experiments, in which it can be observed that the main compound formed after the microwave radiation is the double perovskite precursor phase SrMoO_4_ (PDF: 085-0586), while in experiments A and B, hematite Fe_2_O_3_ (PDF:024-0072) was also observed. As the temperature reached 220 °C in A and B, it seems that high temperature reached (>170 °C), by using microwave radiation, enhanced the generation of the phases SrMoO_4_ and Fe_2_O_3_, the former being the most abundant. Notably, in these series of experiments pH was kept at 2.5 while reaction time was either 30 min (A and C experiments) or 90 min (B and D experiments). It is likely that, by reducing the reaction temperature using microwave radiation, precursor phases such as SrMoO_4_ might be formed almost selectively, without the presence of hematite (Fe_2_O_3_). Nevertheless, another precursor phase, namely SrFeO_2.5_, was not observed in the product of these experiments.

**Figure 2:**
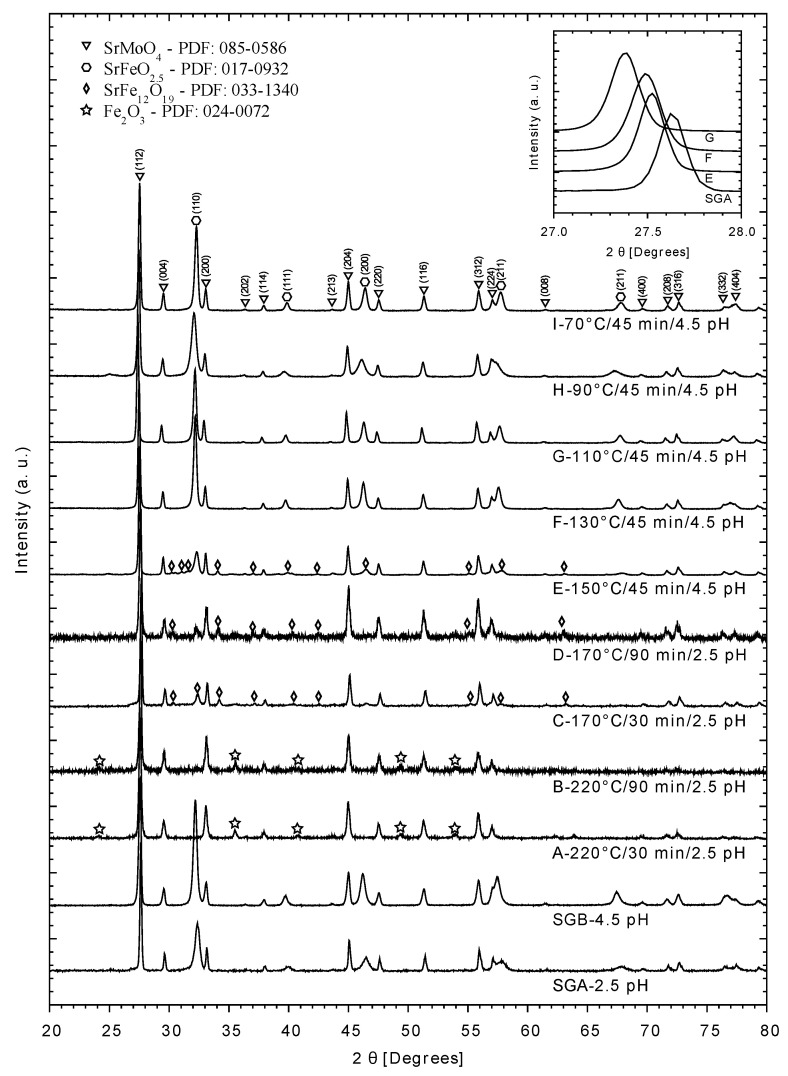
XRD patterns of powders obtained after calcination for reference experiments (SGA and SGB) and those in which microwave radiation was used (A, B, C, D, E, and F). Bragg reflections attributed to the metallic precursors are highlighted (SrMoO_4_ (PDF: 085-0586), SrFeO2.5 (PDF: 017-0932), SrFe_12_O_19_ (PDF: 033-1340) and Fe_2_O_3_ (PDF: 024-0072)).

Concerning the comparison of the products from traditional and microwave assisted hydrothermal methods, the crystallographic results from XRD in Figure 2 show the pattern of the final reaction after the calcination stage at 900 °C. SGA and SGB correspond to the reference SMFO obtained by the Sol-Gel method, while those with the influence of microwaves were the A to I experiments. According to this diffractogram, the precursor phases SrMoO_4_ (PDF: 085-0586) and SrFeO_2.5_ (PDF: 017-0932) were obtained in both experiments SGA and SGB, with the difference that, in the latter, the SrFeO_2.5_ phase has a higher counting number in its Bragg reflections compared to former. During the Sol-Gel microwave assisted method, increasing the pH promoted the formation of SrFeO_2.5_. This result was used to perform experiments E and F, in order to obtain a higher purity SMFO (lack of hematite) after the reduction stage. The difference between these two experiments was the temperature reached during radiation. A higher temperature was reached in E (150 °C) as compared to F (130 °C). In both experiments, both precursor phases were observed, SrMoO_4_ and SrFeO_2.5_. Experiments C and D have the precursor phases, and though the presence of hematite is not observed, there is the presence of traces of a macro-oxide structure SrFe_12_O_19_ that is observed only at high temperatures (≥150 °C). It should be mentioned that this compound did not affect the final product of SMFO.

Regardless of the reaction time (30 min in C and 90 min in D), precursor phases SrMoO_4_, SrFeO_2.5_, and macro-oxide structure SrFe_12_O_19_ (ascribed to an intermediate phase) led to the synthesis of high purity SFMO [25] that is observed from the XRD spectrum. Lower temperatures were tested using the better conditions of the microwave-assisted synthesis in order to get a general idea of how temperature affects the synthesis process (samples G to I). The diffractograms show the formation of the precursor phases SrMoO_4_ and SrFeO_2.5_ as in samples E, F, and G, with slight shifts to the left if the SGA is taken as a reference. In Figure 2, an enlargement of the principal reflection of SrMoO_4_ has been made (shown in the upper right of the graph), in order to observe these displacements.

**Figure 3:**
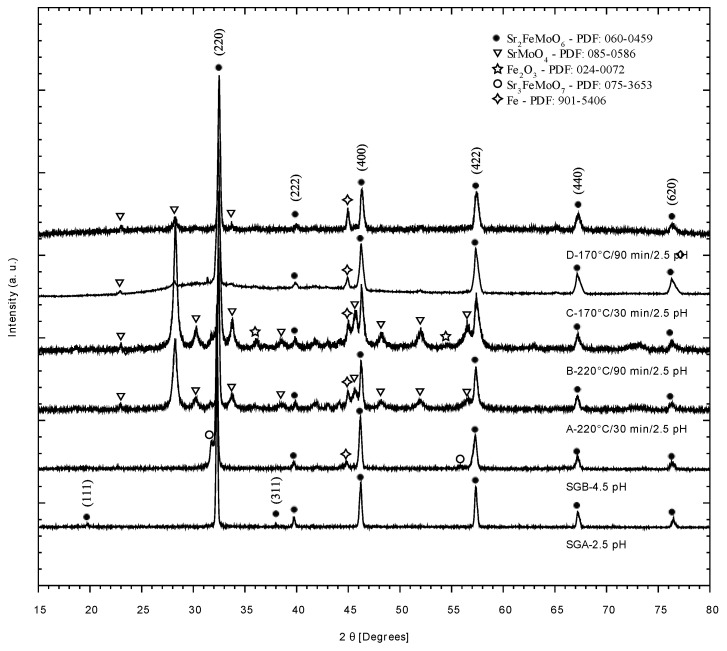
XRD patterns after the reduction process. Bragg reflections attributed to metallic precursor are highlighted (Sr_2_FeMoO_6_ (PDF: 060-0459), SrMoO_4_ (PDF: 085-0586), Fe_2_O_3_ (PDF: 024-0072), Sr_3_FeMoO_7_ (PDF: 075-3653), and Fe (PDF: 901-5406)).

Suffice it to say that the products shown in Figure 2 were not reduced yet. Hence, once the reduction stage performed at 1200 °C under Ar/H_2_ atmosphere (see Methods section) the final product was characterized by XRD analysis, and the results are depicted in Figure 3. Experiments A, B, and D are considered to have failed, in the sense that it is possible to identify the SrMoO_4_ precursor phase, along with traces of Fe (PDF: 901-5406). The fact that all precursor phases are observed in experiments A to D, even after the reduction stage, indicates that double perovskite Sr_2_FeMoO_6_ might not be formed if pH and temperature are set at higher or lower value, respectively. Furthermore, the reaction time is related to the formation of the double perovskite, the optimum value being 45 min. Experiments F to I have the same precursor phases as SGA and SGB, with the difference that for the SrFeO_2.5_ phase there is a variation in its intensities. Undoubtedly, the temperature, the time of exposure to microwaves, and the pH are important factors for the formation of the precursor phases of SMFO, where unwanted compounds (such as traces of Fe, SrFe_12_O_19_, and Fe_2_O_3_) are diminished or eliminated.

Based on these results, it can be inferred that, by reducing the reaction temperature, using microwave radiation might lead to better crystallographic results of the double perovskite product. In this regard, three additional experiments were performed for which their final product (after calcination and reduction stage) was analyzed by XRD similarly to those of the previous experiments (SGA, SGB, E, and F).

**Figure 4:**
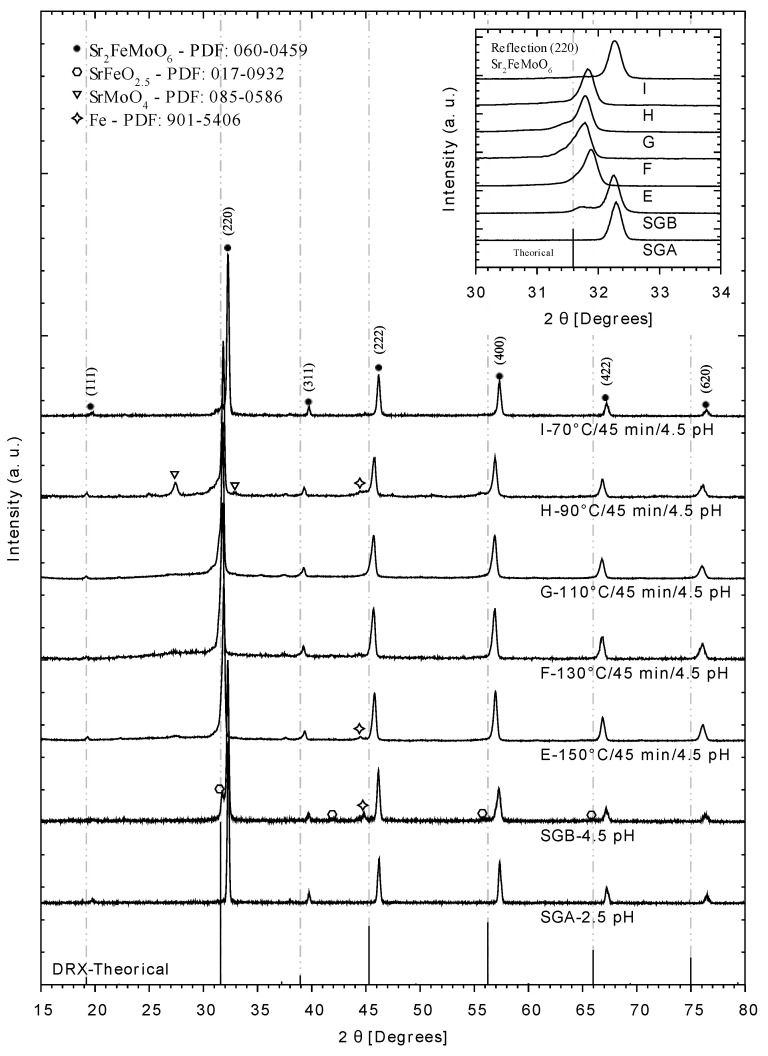
XRD patterns of obtained double perovskite Sr_2_FeMoO_6_ after the reduction process referred to the theoretical Bragg reflections (dotted lines) [27]. The condition labels refer to reaction temperature, reaction time, and pH, respectively.

Figure 4 shows the comparison of the most significant XRD pattern for the best double perovskite products, in which those related to lower reaction temperature, namely G, H, and I experiments, are also depicted. All these experiments, except the references SGA and SGB, used microwave radiation during synthesis, leading to different reaction temperatures. However, pH 4.5 and reaction time (45 min) were the same. The gray dashed line indicates the theoretical Bragg reflection (2θ ≈ 31.588) for ideal double perovskite, and it is used for further discussion of the experimental results. The Bragg reflection of the reference experimental products (SGA (2θ ≈ 32.295) and SGB (2θ ≈ 31.259)), as well as that from I (2θ ≈ 32.270), H (2θ ≈ 31.825), and E (2θ ≈ 31.887), shifted to slightly higher values than the theoretical red line reflection. On the other hand, the Bragg reflection of F (2θ ≈ 31.780) and G (2θ ≈ 31.791) experiments were closer to the theoretical one. These results indicate a strong dependence of the structural features of the double perovskite product that is related to the physical stress of the solid. The fact that the Bragg reflection shifts to the right indicates that the crystal lattice is compressed [26], which is due to a structural defect caused by the physicochemical and thermal environment, regardless of using microwave radiation, but keeping a rather low reaction temperature. It can be said that the structural defect is not related to the presence of the microwaves, but the double perovskite crystallite size is, as it is decreasing when microwaves are used compared to the normal Sol-Gel method. Additionally, the main reflections for SMFO, (220), (400), and (422), show some asymmetry (see Figure 4) which correspond to experiments F, G, and H. This can be attributed to the formation of the tetragonal phase (I4/m) of the double perovskite. In the upper right part, a more significant diffraction enlargement has been made, so that the aforementioned displacements can be observed in detail.

Treating the XRD data by the size-strain plot (SSP) method revealed that, for all double perovskite products (experiments E and F, as well as SGA and SGB), the data fit to the model described in Section 2.4. This is related to both the widening and stress in crystallite. The samples reached values above 0.98 and 0.97 for R^2^ for reference SGA and SGB, respectively, while for the products using microwave radiation the range were 0.99 for E, 0.96 for F and G, and 0.98 for H and I. In the end, this method allows us to estimate the average crystallite size that is summarized in Table 2 and depicted in Supplementary Material Figure S1.

There is no straight tendency of the reaction temperature, but, as a general remark, using microwave radiation leads to smaller particle size than the non-assisted microwave technique. This result, together with that previously mentioned from the XRD analysis, indicates that, regardless of the reaction temperature, microwaves promote less compression forces in the crystal lattice, which results in smaller crystallite size and shifting (to the right side) of the theoretical Bragg reflections. In the end, it seems that the effect of microwave radiation when obtaining double perovskite lies in the advantage of the thermal equilibrium reached in a rather short reaction time (SGA and SGB experiments were followed during 3 h), whereas the size and quality of the crystal lattice have slight differences in both synthesis methods.

### 3.2. Scanning Electron Microscopy and Energy Dispersive X-ray Spectroscopy

**Figure 5:**
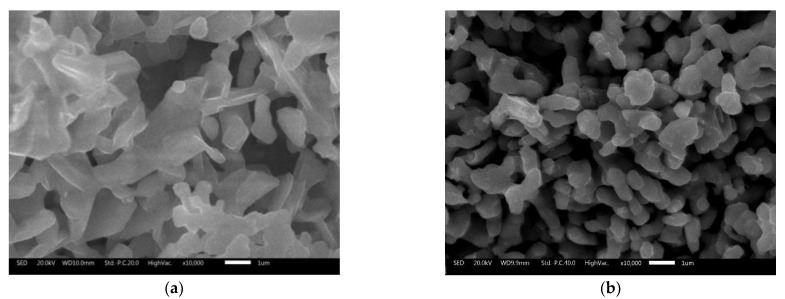
SEM images of perovskite products; (**a**) SGB experiment; (**b**) G experiment. The samples are polycrystalline with interconnected pores in its structure and accumulation of particles (crystalline aggregates).

The micrographs obtained by SEM for the SGB product of non-assisted microwave synthesis and experiment G by microwave synthesis are shown in Figure 5. It can be observed that all the samples have interconnected pores in its structure and accumulation of particles (crystalline aggregates).

**Figure 6:**
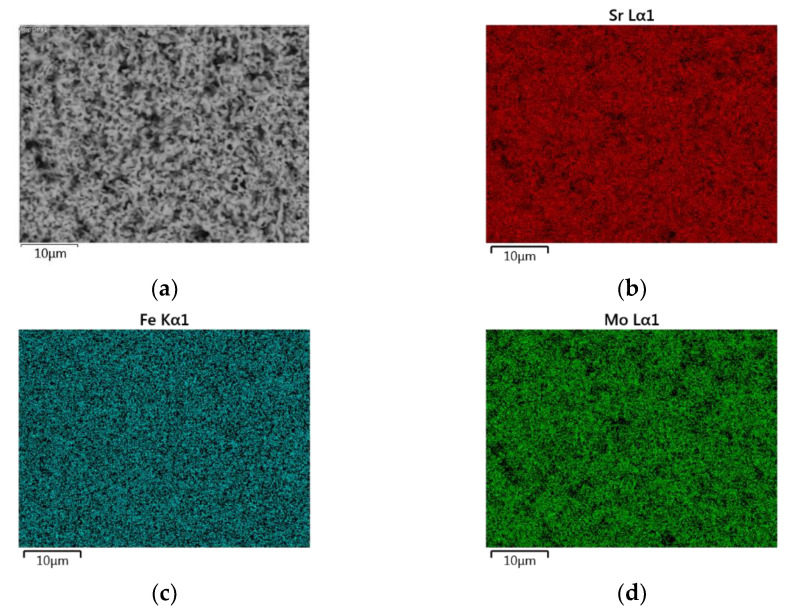
EDS mapping for a section of the sample SGA after the reduction process (**a**). A uniform distribution of strontium (**b**), iron (**c**), and molybdenum (**d**) atoms can be observed.

**Figure 7:**
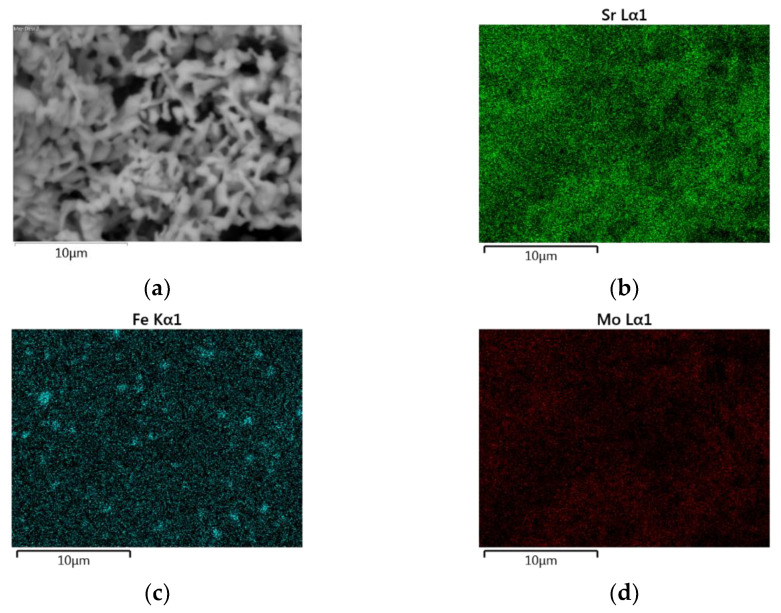
EDS mapping for a section of the sample SGB after the reduction process (**a**). A uniform distribution of strontium (**b**) and molybdenum (**d**) atoms can be observed while a non-uniform distribution of iron (**c**) atoms is obtained.

**Figure 8:**
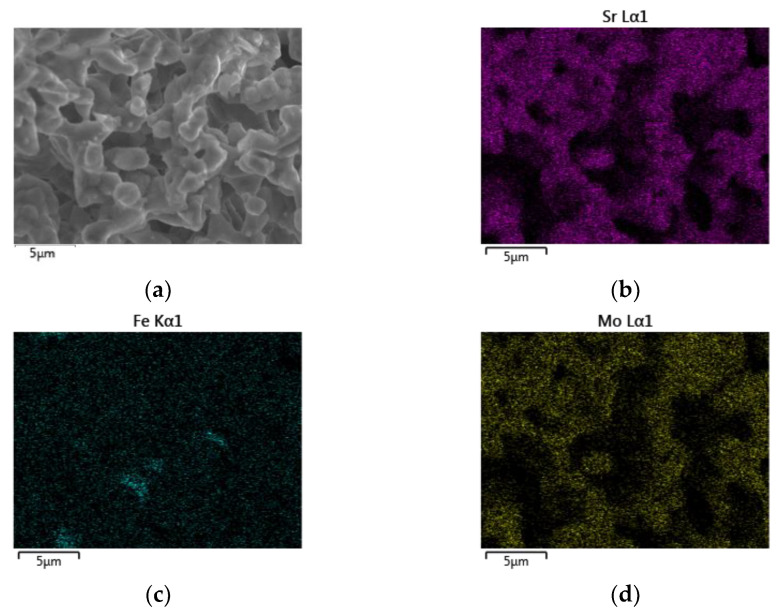
EDS mapping for a section of the sample E after the reduction process (**a**). A homogeneous distribution of strontium (**b**) and molybdenum (**d**) atoms can be observed while a non-uniform distribution of iron (**c**) atoms is obtained.

**Figure 9:**
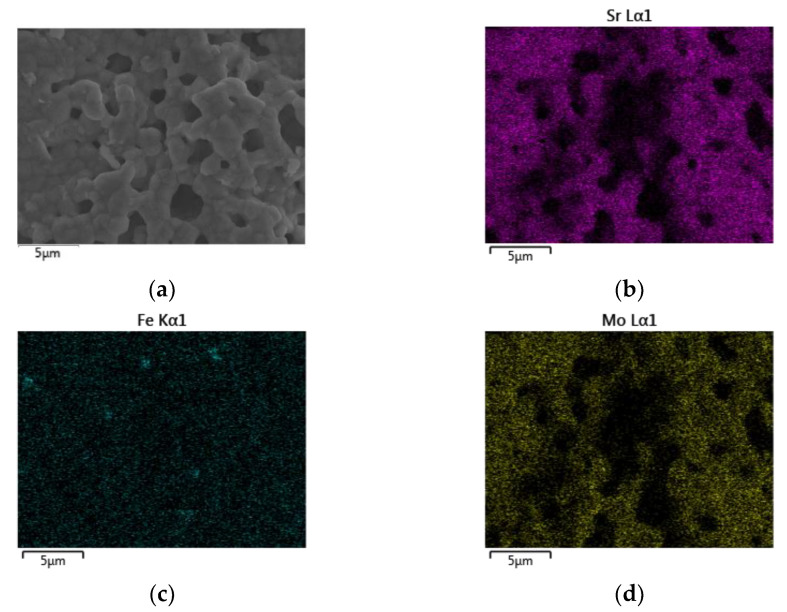
EDS mapping for a section of the sample F after the reduction process (**a**). A homogeneous distribution of strontium (**b**) and molybdenum (**d**) atoms can be observed while a non-uniform distribution of iron (**c**) atoms is obtained.

The EDS mapping images of the SFMO from SGA, SGB, E, and F experiments are shown in Figure 6, Figure 7, Figure 8 and Figure 9, respectively. Starting with experimental reference SGA (Figure 6), it can be observed that a uniform distribution of strontium, iron, and molybdenum metal atoms are obtained; this is rather similar to that obtained in experiment SGB (Figure 7), except for the iron atoms, as an apparent uneven concentration of these is observed in some specific areas (spots). In the case of SFMO obtained with microwaves assisted method (experiments E and F), mapping shows (Figure 8 and Figure 9, respectively) that there is a homogeneous metal atom distribution for the cases of Sr and Mo, but a slightly more located distribution for Fe atoms (dots rather than spots).

### 3.3. Thermogravimetric Analysis and Differential Scanning Calorimetry

**Figure 10:**
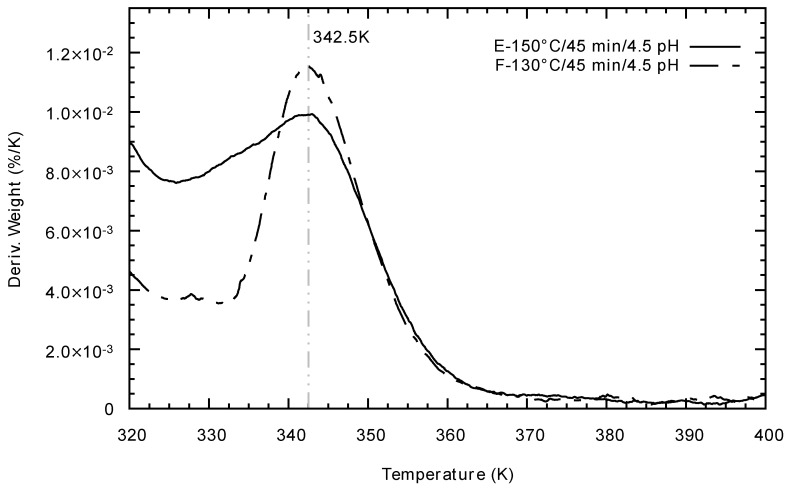
Differential thermogravimetric analysis for experiments E and F. This change in intensity is an indication that there are fewer Weiss domains in the perovskite obtained by experiment E than experiment F.

Curie Temperature could be determined by Thermogravimetric Analysis (TGA) (for details please refer to the Materials and Methods section). Figure 10 shows the differential thermogravimetric analysis for the experiments E and F after the reduction process. Both samples have the same Tc; this Tc value corresponds to experiments reported in the literature [28], although sample F presents a more intense signal, and the loss of its ferromagnetic properties begins at higher temperature compared to sample E. This change in intensity is an indication that there are fewer Weiss domains in the double perovskite obtained by experiment E than experiment F, which is in line with what was reported in [29].

## 4. Discussion

According to the results obtained by X-ray diffraction, high pressures and temperatures inside the reactor, i.e., more than 40 bar and 150 °C, respectively, lead to iron oxide phases formation and the insulating phase of SrTiO_4_, as shown in Figure 3. Temperature conditions between 90 C and 150 °C induce a shift in the diffractograms to their theoretical X-ray diffraction, however, only the temperature of 130 °C, corresponding to experiment F, produced a pure phase of SFMO (Figure 4). This result indicates the formation of a more stable gel, where the creation of metallic citrate is stimulated. Similarly, the crystallite size suffers a decrease under the conditions of experiment F. In agreement with the decrease in crystallite size, experiment F generates an intense response in the phase transition compared to experiment E (Figure 10), in which its crystallite size is comparable to those obtained in traditional Sol-Gel experiments. This change in intensity is an indication that there are more Weiss domains in the double perovskite obtained by experiment F. According to literature reported data [16,18], the shift to the right of the Bragg’s reflections (Figure 4) is related to a non-ordered Fe/Mo site distribution, where more antisites Fe-O-Fe are found within the structure. Then, when the SFMO diffractogram resembles the theoretical reference pattern, a more ordered Fe/Mo relationship would be expected; from the set of experiments carried out, the sample F would be the closest to the theoretical one.

## 5. Conclusions

The physical properties of SFMO double perovskite can be controlled via the micro-wave-assisted hydrothermal process in the early stages of the Sol-Gel synthesis. The change in crystallite size is, in part, a consequence of the microwave radiation promoted by the increase in temperature and pressure in the reactor. Heating by this latter method is uniform, which enhances the homogeneous diffusion and nucleation during synthesis, giving better Fe/Mo order as compared to Sol-Gel non-assisted. In fact, the change in crystallite size is a decrease, but keeps good crystallinity of the double perovskite product, also promoted by the carefully performed calcination and reduction stages. The lowering of the crystallite size in SFMO has direct consequences on the Tc. By the radiation of microwaves, the Tc value was higher with respect to the normal Sol-Gel synthesis, from 335 K to 342.5 K. This, logically, affects the magnetic behavior of the SFMO double perovskite.

## Figures and Tables

**Table 1:** Experimental conditions for the Sol-Gel assisted by microwave.

Experiment	Temperature (°C)	Pressure (Bar)	Reaction Time (min)	pH
A	220	80	30	2.5
B	220	80	90	2.5
C	170	46	30	2.5
D	170	45	90	2.5
E	150	42	45	4.5
F	130	30	45	4.5
G	110	35	45	4.5
H	90	33	45	4.5
I	70	20	45	4.5

**Table 2:** Calculated crystallite size of double perovskite from SGA and SGB reference experiments, and those from microwave assisted technique E, F, H, and I. The average values were obtained by the SSP method. The complete statistical data from linear fit is presented in Supplementary Materials Table S1.

Experiment	Crystallite Size (nm)	Temperature (°C)
SGA	420	80
SGB	345	80
E	378	150 ^1^
F	310	130 ^1^
G	401	110 ^1^
H	352	90 ^1^
I	318	70 ^1^

^1^ Microwave heating.

## Data Availability

The data presented in this study are available on request from the corresponding author.

## Supplementary Materials

The following are available online at https://www.mdpi.com/article/10.3390/ma14143876/s1, Figure S1: Size-Strain Plot: (a) Data and linear fit of the SGA experiment, (b) Data and linear fit of the SGB experiment, (c) Data and linear fit of the E experiment, (d) Data and linear fit of the F experiment, (e) Data and linear fit of the G experiment, (f) Data and linear fit of the H experiment, (g) Data and linear fit of the I experiment, Table S1: Calculated crystallite size of double perovskite from SGA and SGB reference experiments and that from microwave assisted technique E, F, H, and I. The average values were obtained by the SSP method.
