# Supplementary material for: Sol-Gel Synthesis of the Double Perovskite Sr2FeMoO6 by Microwave Technique
Source: Materials (Basel). 2021 Jul 12;14(14):3876. doi: 10.3390/ma14143876 (PMC8305548; doi:10.3390/ma14143876)
Supplement: Supplementary file 1 [file materials-14-03876-s001.zip › materials-1239220-SI.pdf]

materials

Supplementary Materials

Sol-Gel Synthesis of the Double Perovskite Sr_2FeMoO_6 by Microwave Technique

Jesús Valdés [1], Daniel Reséndiz [1], Ángeles Cuán [1,*], Rufino Nava [1], Bertha Aguilar [2], Carlos M. Cortés-Romero [3] and Oracio Navarro [2]

[1] División de Estudios de Posgrado, Facultad de Ingeniería, Centro Universitario, Universidad Autónoma de Querétaro, Querétaro 76010, Mexico; jesus.valdes@uaq.mx (J.V.); daniel.m.resendiz@gmail.com (D.R.); rufino@uaq.mx (R.N.)
[2] Unidad Morelia, Instituto de Investigaciones en Materiales, Universidad Nacional Autónoma de México, Antigua carretera a Pátzcuaro No. 8701, Col. Ex Hacienda de San José de la Huerta, Morelia 58190, Mexico; baguilar@iim.unam.mx (B.A.); navarro@unam.mx (O.N.)
[3] Facultad de Química, Centro Universitario, Universidad Autónoma de Querétaro, Querétaro 76010, Mexico; carlos.cortes@uaq.mx
* Correspondence: maria.delosangeles.cuan@uaq.mx; Tel.: +52-44-2192-1200 (ext. 65424); Fax: +52-44-2192-1200 (ext. 6006)

Citation: Valdés, J.; Reséndiz, D.; Cuán, A.; Nava, R.; Aguilar, B.; Cortés-Romero, C. M.; Navarro, O. Sol-Gel Synthesis of the Double Perovskite Sr_2FeMoO_6 by Microwave Technique. *Materials* **2021**, *14*, 3876. https://doi.org/10.3390/ma14143876

Academic Editor: Efrat Lifshitz

Received: 13 May 2021
Accepted: 24 June 2021
Published: 12 July 2021

Publisher's Note: MDPI stays neutral with regard to jurisdictional claims in published maps and institutional affiliations.

Copyright: © 2021 by the authors. Submitted for possible open access publication under the terms and conditions of the Creative Commons Attribution (CC BY) license (http://creativecommons.org/licenses/by/4.0/).

Crystallite Size Calculation with the *Size-Strain Plot* Model.

The model for Size-Strain Plot contemplates the Bragg's reflections broadening as a contribution of the crystallite size and strain, contemplating the anisotropic nature of the strain. This method it is assumed that the crystallite size profile is described by a Lorentzian function, while the strain in the lattice is described by a Gaussian function, the convolution of both functions describes the broadening of the Bragg's reflections. For this method, the strain variable, does not have a dependence on θ, and there is less wight given to data from reflections at high angles, where the precision is typically lower. In this way, one as:

$$(d_{hkl}\beta_{hkl}\cos(\theta))^2 = \frac{K}{D}(d_{hkl}^2\beta_{hkl}\cos(\theta)) + \left(\frac{\varepsilon}{2}\right)^2$$

where K is a constant that describes the geometrical shape of the crystallite, in this work we assume spherical crystallites, this gives the value for K = 3/4. The subscripts *hkl* are the Miller indices, d is the interplanar distance, β the Full Width at Half Maximum of the convolution of the Lorentzian ang Gaussian function, θ represents the Bragg's reflection in 2θ, $\mathcal{E}$ is the strain, and finally D is the diameter of the crystallite, the size.

Similarly, to the Williamson-Hall method the term $(d_{hkl}\beta_{hkl}\cos(\theta))^2$ is plotted with respect to $(d_{hkl}^2\beta_{hkl}\cos(\theta))$. The Crystallite size D is determined form the slope of the linearly fitted data, and the root of the $(d_{hkl}\beta_{hkl}\cos(\theta))^2$ axis intercept gives the strain.

Table S1 indicates the Crystallite size for the SGA, SGB, E, F, G, H and I experiments, as well as the statistical data from the linear fit. The Root Mean Squared Error (RMSE) was obtained from the linear fit and was used to obtain the Standard Deviation of the Crystallite size. Positive strain indicates that the crystal is at tensile strain and negative strain indicates that the crystal is at compressive strain.

Table S1. Calculated crystallite size of double perovskite from SGA and SGB reference experiments and that from micro-wave assisted technique E, F, H, and I. The average values were obtained by the SSP method.

Experiment	Crystallite size (D) (nm)	Strain <ε> no units $\times 10^{-8}$	RMSE SSP ($\times 10^{-8}$)	Standard Deviation Crystallite size (nm)	Temperature (°C)
SGA	420	1.93	1.90	0.005	80
SGB	345	2.12	5.13	0.008	80
E	378	7.18	2.08	0.003	150[1]
F	310	6.20	2.98	0.003	130[1]
G	401	12.20	5.55	0.011	110[1]
H	352	6.02	5.00	0.008	90[1]
I	318	−0.37	4.22	0.005	70[1]

[1] Microwave heating.

Figure S1 shows the Size Strain Plots for the SGA, SGB, E, F, G, H and I experiments.

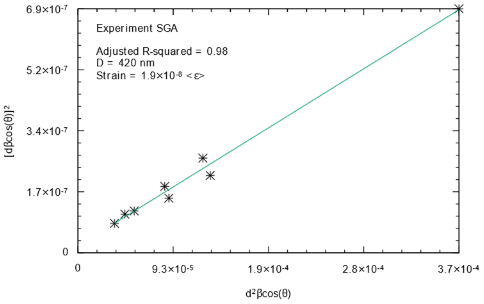

(**a**)

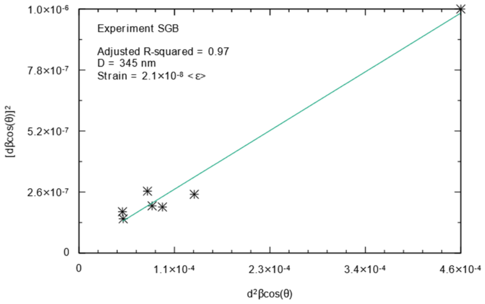

(**b**)

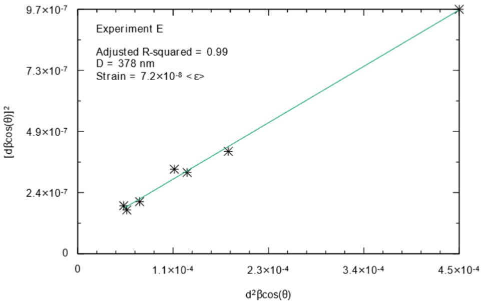

(**c**)

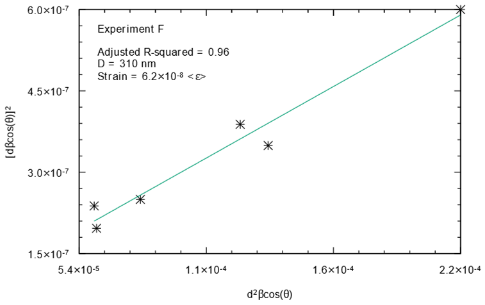

(**d**)

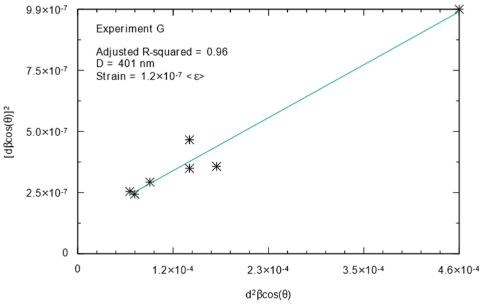

(**e**)

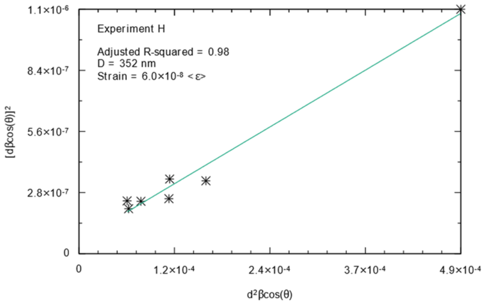

(**f**)

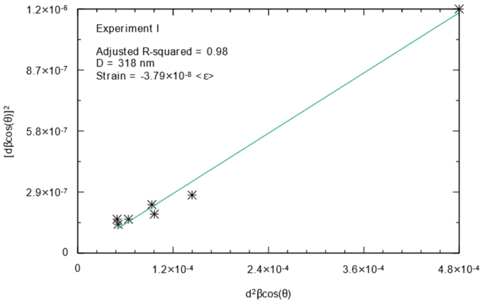

(**g**)

Figure S1. Size-Strain Plot: (**a**) Data and linear fit of the SGA experiment, (**b**) Data and linear fit of the SGB experiment, (**c**) Data and linear fit of the E experiment, (**d**) Data and linear fit of the F experiment, (**e**) Data and linear fit of the G experiment, (**f**) Data and linear fit of the H experiment, (**g**) Data and linear fit of the I experiment.
